# L-carnitine promotes liver regeneration after hepatectomy by enhancing lipid metabolism

**DOI:** 10.1186/s12967-023-04317-x

**Published:** 2023-07-20

**Authors:** Xi Zhou, Guobin Huang, Lu Wang, Yuanyuan Zhao, Junbo Li, Dong Chen, Lai Wei, Zhishui Chen, Bo Yang

**Affiliations:** Institute of Organ Transplantation, Tongji Hospital, Tongji Medical College, Key Laboratory of Organ Transplantation, NHC Key Laboratory of Organ Transplantation, Key Laboratory of Organ Transplantation, Huazhong University of Science and Technology, Ministry of Education, Chinese Academy of Medical Sciences, No.1095 Jiefang Avenue, Wuhan, 430030 P.R. China

**Keywords:** L-carnitine, Lipid metabolism, Liver regeneration

## Abstract

**Background:**

Lipid metabolism plays an important role in liver regeneration, but its regulation still requires further research. In this study, lipid metabolites involved in mouse liver regeneration at different time points were sequenced and analyzed to study their influence on liver regeneration and its mechanism.

**Methods:**

Our experiment was divided into two parts. The first part examined lipid metabolites during liver regeneration in mice. In this part, lipid metabolites were sequentially analyzed in the livers of 70% mouse hepatectomy models at 0, 1, 3and 7 days after operation to find the changes of lipid metabolites in the process of liver regeneration. We screened L-carnitine as our research object through metabolite detection. Therefore, in the second part, we analyzed the effects of carnitine on mouse liver regeneration and lipid metabolism during liver regeneration. We divided the mouse into four groups: control group (70% hepatectomy group); L-carnitine group (before operation) (L-carnitine were given before operation); L-carnitine group (after operation)(L-carnitine were given after operation) and L-carnitine + perhexiline maleate (before operation) group. Weighing was performed at 24 h, 36 and 48 h in each group, and oil red staining, HE staining and MPO staining were performed. Tunnel fluorescence staining, Ki67 staining and serological examination.

**Results:**

Sequencing analysis of lipid metabolites in 70% of mouse livers at different time points after hepatectomy showed significant changes in carnitine metabolites. The results showed that, compared with the control group the mouse in L-carnitine group (before operation) at 3 time points, the number of fat drops in oil red staining was decreased, the number of Ki67 positive cells was increased, the number of MPO positive cells was decreased, the number of Tunnel fluorescence positive cells was decreased, and the liver weight was increased. Serum enzymes were decreased. Compared with control group, L-carnitine group (after operation) showed similar trends in all indexes at 36 and 48 h as L-carnitine group (before operation). L-carnitine + perhexiline maleate (before operation) group compared with control group, the number of fat drops increased, the number of Ki67 positive cells decreased, and the number of MPO positive cells increased at 3 time points. The number of Tunnel fluorescent positive cells increased and serum enzyme increased. However, both liver weights increased.

**Conclusion:**

L-carnitine can promote liver cell regeneration by promoting lipid metabolism and reduce aseptic inflammation caused by excessive lipid accumulation.

## Introduction

Liver regeneration is important for recovery after major liver resection, relative liver transplantation and other diseases such as acute and chronic hepatitis. Under normal physiological condition, only a handful of hepatocytes are in proliferation state, but exhibit strong regenerative ability once stimulated, either through tissue damage or tissue injury. Following tissue injury, hepatocytes rapidly enter into the early stage of DNA synthesis. Compared with other parenchymal organs such as the kidney, heart and lungs, the liver possesses strong regenerative capacity, mainly because hepatocytes are stable cells. Although they do not proliferate significantly under normal conditions, the process of liver regeneration is initiated if the liver volume decreases or hepatocytes are injured. Due to hepatocytes’ remarkable regenerative ability [1] the liver volume and function return to normal in approximately two weeks after 70% hepatectomy in mouse and four months in humans.

Early after liver resection, regeneration ability is insufficient due to the shortage of liver cells and liver volume, high pressure in the portal vein, long sustained time and small liver syndrome that is characterized by postoperative liver dysfunction, high blood bilirubin with prolonged cholestasis, blood coagulation disorders, portal hypertension, and the emergence of serious ascites. Small liver syndrome has a very high mortality rate and requires liver transplantation [2–4]. Liver regeneration is especially crucial for relative liver transplantation, where both the donor and the recipient are at risk of small liver syndrome, and the recipient can potentially develop cold and warm ischemia injury. Moreover, it can seriously affect the liver’s ability to regenerate [5]. Although the regulation of liver regeneration has been widely researched, its detailed mechanism remains not fully elucidated.

Liver is one of the most important organs for lipid metabolism. Hepatocytes contain numerous of enzymes involved in lipid metabolism. In diabetics and during the state of hunger, liver cells can rapidly use fat to supply energy and export ketone bodies to other organs [6, 7]. After liver resection, glucocorticoids increase due to preoperative and postoperative fasting and surgical stress, resulting in a lower glucose utilization rate. However, liver regeneration requires a large amount of energy, thus hepatocytes utilize fat mobilization, absorb a large amount of fat, and reuse stored fat to fulfill energy demands [8, 9]. Although the vast reserves of stored fat can provide a lot of energy for liver regeneration, excessive lipid accumulation and lipid peroxidation can induce apoptosis of liver cells and cause aseptic inflammation, affecting liver function [10]. The role of lipid metabolism in liver regeneration has not been conclusioned.

L-carnitine is an endogenous molecule involved in fatty acid metabolism. L-lysine and L-methionine are used as substrates in human biosynthesis [11]. In the cell, fatty acids enter the mitochondria as acylcarnitine via L-carnitine and are metabolized by beta-oxidation. In this pathway, energy and acetyl-coA are produced, each cycle of the fatty acid chain is shortened by two carbons, and the released acetyl-coA then enters the citric acid cycle, during which further energy is produced [12]. Lipoacylcarnitine transferase is the rate-limiting enzyme of β oxidation. When carnitine deficiency is congenital or acquired, beta oxidation is significantly inhibited [13]. L-cartine, because of its function can promote the lipid metabolism has been applied to the treatment. Including weight loss [14], heart disease [15], kidney disease [13] and cerebral disease [16, 17]. The liver is an important organ for lipid metabolism, L-carnitine is also used in various liver diseases, including non-alcoholic fatty liver disease [19], cirrhosis [20],Liver cancer [21] and chronic liver disease [12]. The role and mechanism of L-carnitine in liver regeneration have not been studied.

In this study, 70% liver resection model was used to observe the process of liver regeneration in mice, and lipid metabolites were sequentially sequentially conducted to identify metabolites with significant changes in liver regeneration, whether there were carnitine lipid metabolites changes, and to verify the role and specific mechanism of L-carnitine on liver regeneration.

## Material and methods

### Animals

**Fig. 1:**
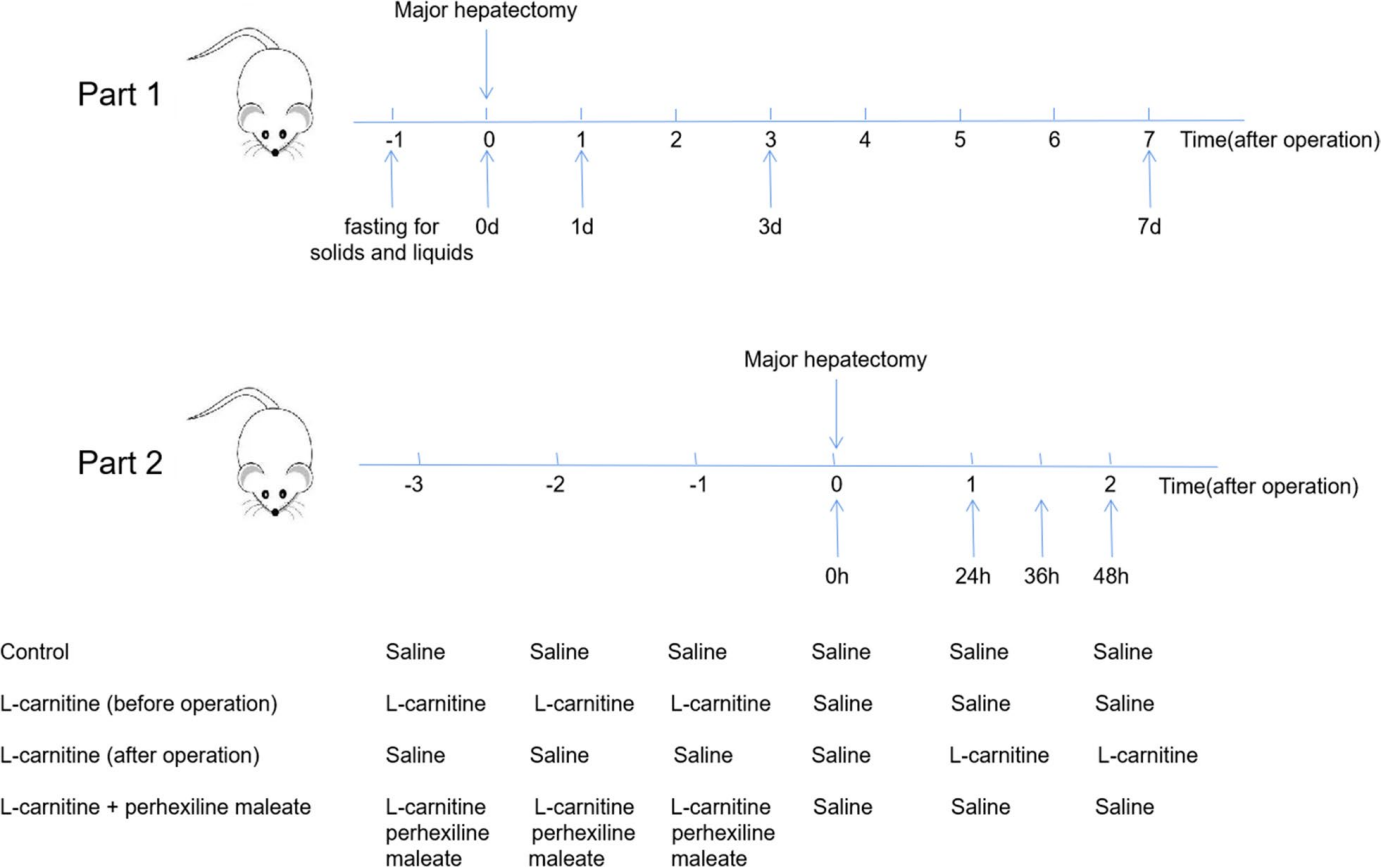
Mouse treatment diagram

60 male C57BL/6J mouse (6–8 weeks old) were purchased from Beijing Vital River Laboratory Animal Technology Co., Ltd. (Beijing, China). The animals were kept at Huazhong University of Science and Technology’s Animal Facility at Tongji Hospital and Tongji Medical College. Huazhong University of Science and Technology’s Tongji Medical College Institutional Animal Care and Use Committee approved all animal experiments(TJH-01008). We followed all guidelines and regulations set by the Chinese Council on Animal Care. All mice were sacrificed by anesthesia and bloodletting. Our experiment was divided into two parts. The first part was the analysis of lipid metabolites in most mice after hepatectomy. In this section twelve mice were divided into four groups (0d, 1d, 3d and 7d) with 3 mice in each group. All four groups of mice underwent most of the hepatectomy (24 h water fasting before surgery), and samples were taken after 0, 1, 3, and 7 days of postoperative anesthesia (intraperitoneal injection of 1% pentobarbital sodium (7 µL/g body weight), respectively (24 h water fasting before specimen collection). The second part is the effect of carnitine on liver regeneration and lipid metabolism in the process of liver regeneration. In this part, mouse were divided into four groups (12 mice in each group) : L-carnitine (before operation) group: L-carnitine (200 mg/kg day) was given by gavage 3 days before operation; L-carnitine (after operation) group: L-carnitine (200 mg/kg day) was given by gavage 1 day after operation;L-carnitine + perhexiline maleate group: L-carnitine (200 mg/kg day) and carnitine acyl transferase I inhibitor (perhexiline maleate (80 mg/kg day)) were given by gavage 3 days before operation, and control group: Saline was given by gavage 3 days before operation and one day after surgery. Mice in each group were divided into four time points (oh, 24 h, 36 h, 48 h), 3 mice at each time point). All mice in each group underwent subtotal hepatectomy at 0 and 24 h, respectively. Samples were taken after 36-hour and 48-hour anesthesia (intraperitoneal injection of 1% pentobarbital sodium (7 µL/g body weight)) (24 h water fasting before specimen collection) (Fig. 1).

### Major hepatectomy in mouse

First, 10% chloral hydrate was administered intraperitoneally. After abdominal disinfection, a vertical abdominal midline incision was made under xiphoid process. The epidermis, muscularis and peritoneum were cut open and the abdominal muscles were opened to expose the liver. The ligaments associated with the liver were separated using eye scissors, and the middle and left lateral lobe of the liver were successively ligated and excised, while preserving the gallbladder. The remaining liver tissue and abdominal contents were collected, and the abdomen was sutured sequentially. The mouse were deprived of food and water for 6 h after surgery. Glucose injection (5%) was administered subcutaneously to supplement fluid loss during evaporation, bleeding, and drainage, and as nutrition. The injection was rewarmed in an incubator at 37 ℃ for 6 h. The animals were sacrificed at different points in time after operation based on the time point requirements of each group. The weight of the regenerated liver in each group was measured and recorded. The liver was partially frozen and partially fixed with paraformaldehyde.

### Serum biochemistry assay

In order to extract the plasma, the mouse’s blood was first chilled on ice (30 min) and then centrifuged at 8000 Revolutions Per Minute (rpm) for 15 min at room temperature (25 ℃). A standard automatic analyzer (Mindray BS-200, Shenzhen) was used to measure serum levels of alanine transaminase (ALT) and aspartate transporter (AST).

### Hematoxylin and eosin (H&E) staining

After 6 h of ischemia, the liver was then embedded in paraffin after being fixed in 4% paraformaldehyde and dehydrated employing an increasing gradient of ethanol concentration. Afterwards, 4-mm-thick sections were made and stained with H&E.

### Oil red staining of tissue frozen sections

First, the tissue sample was cut to an appropriate size (approximately 2cmx2cmx1cm), embedded with biological glue, wrapped with aluminum foil, and marked with isoprene. Before precooling, the alkane was poured into a small beaker and placed in liquid nitrogen for 30 s. Then, the aluminum foil was put into isopentane and removed after approximately 1 min and put into − 80 ℃, and kept in refrigerator. The wax block was cut into 10 μm thick frozen sections which were treated with 70% ethanol for 1 min and then dyed with oil red for 30 min, treated with 70% ethanol for 1 min, and stained with hematoxylin for 5 min, rinsed with tap water for 2 min and sealed with glycerin.

### LC-MS/MS for analyzing the liver untargeted metabolome after hepatectomy

The prepared samples were thawed on ice. 50 ± 2 mg of each sample was homogenized for 3 min at 30 Hz with cold steel balls. The homogenized centrifuge tub was filled with one microliter of 70% methanol with internal standard extract and the mixture was whirled for five minutes before centrifugation at 12,000 rpm for ten minutes at 4 °C. As soon as the supernatant was centrifuged, 400 mL of the supernatant was poured into the corresponding EP tube and stored overnight at − 20 °C in the refrigerator before being centrifuged at 12,000 r/min for 3 min at 4 °C, and approximately 2000 mL of supernatant was collected in the injection bottle liner for on-board analysis.

### High performance liquid chromatography

A LC-ESI-MS/MS system (UPLC, ExionLC AD, https://sciex.com.cn/; MS, QTRAP® System, https://sciex.com/) was used to analyze the sample extracts. Here are the conditions for the analysis, UPLC: column, Waters ACQUITY UPLC HSS T3 C18 (1.8 μm, 2.1 mm*100 mm); column temperature, 40 °C; flow rate, 0.4 mL/min; injection volume, 2 µL; solvent system, water (0.1% formic acid): acetonitrile (0.1% formic acid); gradient program, 95:5 V/V at 0 min, 10:90 V/V at 10.0 min, 10:90 V/V at 11.0 min, 95:5 V/V at 11.1 min, 95:5 V/V at 14.0 min.

### ESI-QTRAP-MS/MS

This study utilized a tri-quadrupole linear ion trap mass spectrometer (QTRAP®) equipped with an ESI Turbo Ion-Spray interface that operated in positive and negative ion modes and was controlled by Sciex’s Analyst 1.6.3 software, to acquire LIT and triple quadrupole (QQQ) scans. A list of the ESI source operating parameters includes: source temperature 500 ℃; ion spray voltage (IS) 5500 V (positive), − 4500 V (negative); ion source gas I, gas II, curtain gas was set at 55, 60, and 25.0 psi, respectively; the collision gas was high. Polypropylene glycol solutions of 10 and 100 µmol/L were used in QQQ and LIT modes, respectively, for instrument calibration and mass calibration. For each period, specific MRM transformations were monitored in accordance with the metabolites eluted.

### KEGG annotation and enrichment analysis

A KEGG Compound database (http://www.kegg.jp/kegg/compound/) was used to annotate the identified metabolites, which were then mapped to KEGG Pathway database (http://www.kegg.jp/kegg/pathway.html). After determining which pathways had significantly regulated metabolites, MSEA (metabolite sets enrichment analysis) was performed; significance was determined by hypergeometric tests.

### TUNEL assay

A TUNEL Kits (Servicebio, Wuhan, G1501) was used as directed by the manufacturer. The first step in the procedure was to deparaffinize tissue sections with proteinase K. Afterward, membranes were disrupted, the reaction solution was added, and DAPI was used to stain the nuclei. Finally, the sections were imaged under an upright fluorescence microscope (NIKON ECLIPSE C1, Japan), five randomly chosen fields were calculated to determine the proportion of TUNEL-positive cells.

### Myeloperoxidase (MPO) staining

The immunohistochemistry of MPO was conducted using mouse anti-MPO antibodies (Servicebio, Wuhan, GB12224) and HRP-labeled goat anti-mouse antibodies (Servicebio, Wuhan, G23301) along with DAB chromogenic agent kit for histochemistry (Servicebio, Wuhan, GB12224). All procedures were carried out on the basis of the manufacturer’s instructions. After blocking the fixed tissue with serum, we added primary antibodies, secondary antibodies, and developer before staining the nuclei with DAPI. Afterwards, images of the tissue under the microscope were taken and analyzed. Hematoxylin-stained nuclei appeared blue, while MPO expression was marked by a brownish yellow color.

### Ki67 staining

The immunohistochemistry of Ki67 was conducted using mouse anti-Ki67 antibodies (Thermo Fisher, Shanghai, PA5-114437) and HRP-labeled goat anti-mouse antibodies (Servicebio, Wuhan, G23301) along with DAB chromogenic agent kit for histochemistry (Servicebio, Wuhan, GB12224). All procedures were carried out on the basis of the manufacturer’s instructions. After blocking the fixed tissue with serum, we added primary antibodies, secondary antibodies, and developer before staining the nuclei with DAPI. Afterwards, images of the tissue under the microscope were taken and analyzed. Hematoxylin-stained nuclei appeared blue, while Ki67 expression was marked by a brownish yellow color.

### Statistical analysis

Data were analyzed using Prism software (GraphPad, v8.0.2) and expressed as the mean + standard error. One-way ANOVA was used to compare multiples, while unpaired t-tests were used for comparing two groups. P-values < 0.05 were considered statistically significant. In the analysis of LC-MS/MS, we used the statistics function prcomp within R (www.r-project.org) to perform unsupervised principal component analysis (PCA). Before unsupervised PCA, the data were scaled by unit variance. Using hierarchical cluster analysis (HCA), samples and metabolites were presented as heat maps and dendrograms, while Pearson’s correlation coefficients (PCC) between samples were calculated using R’s cor function and presented only as heat maps. R package Complex Heatmap was used for both HCA and PCC analysis. HCA visualizes normalized signal intensities (unit variance scaling) as a color spectrum. For the selected differential metabolites, the significance of differential metabolites between groups was evaluated by VIP > = 1 and absolute log2FC (fold change) > = 1. With R package MetaboAnalystR, VIP values were extracted from OPLS-DA results containing score and permutation plots. For OPLS-DA, the data were log transformed (log2) and centered, and a permutation test (200 permutations) was performed to avoid overfitting.

## Results

### Lipid metabolism after major partial hepatectomy in mouse

**Fig. 2:**
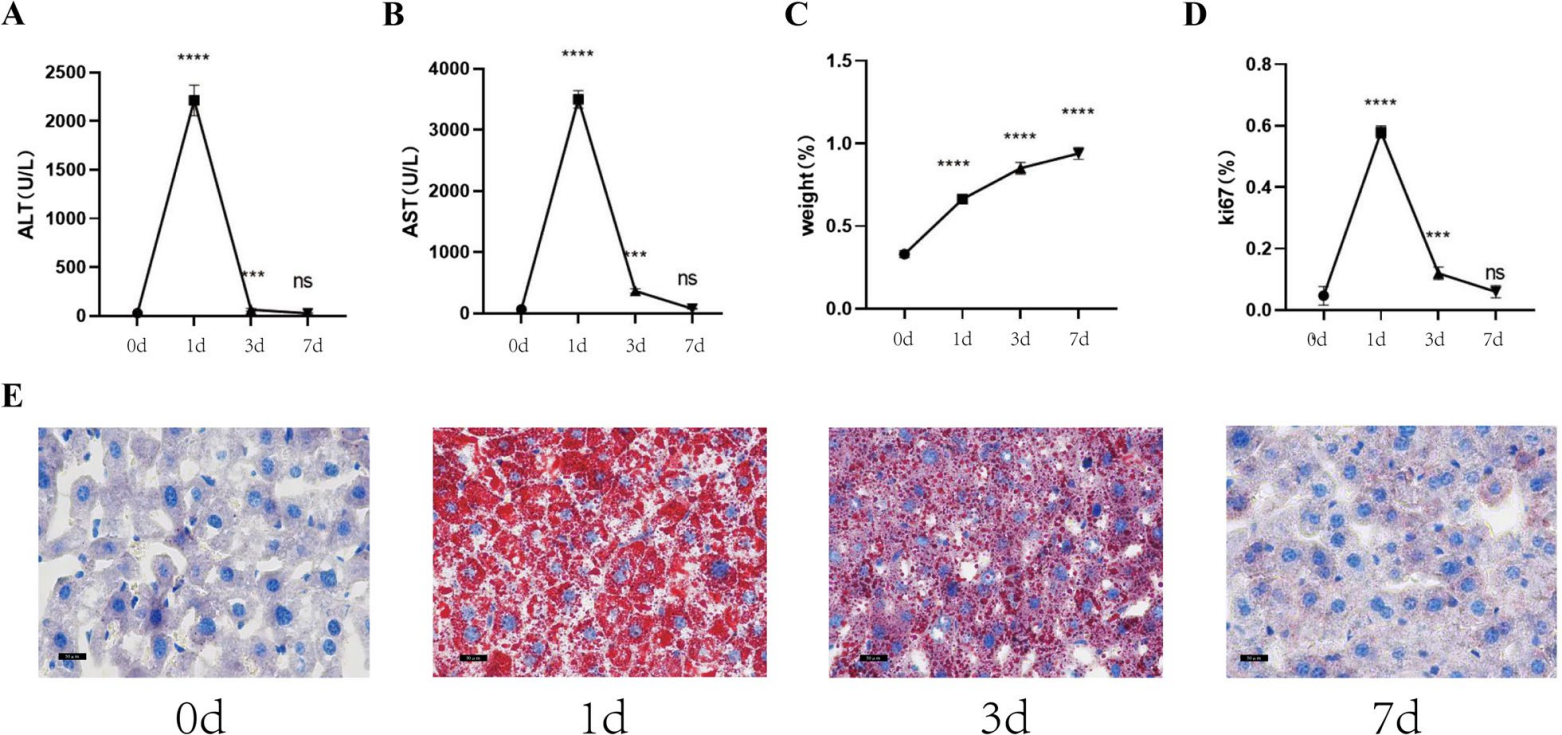
Lipid metabolism after major partial hepatectomy in mouse. **A–B** The liver function indexes (ALT, AST) changed following major partial hepatectomy in mouse. **C** The weight changed after major partial hepatectomy in mouse. **D** The liver regeneration rate changed after major partial hepatectomy in mouse was measured using Ki67. **E** Oil red staining was used to depict the accumulation of lipid droplets (400X). ns p>0.05; ****p<0.0001.

We constructed a model of liver regeneration using major partial hepatectomy in mouse. Serum and liver tissue samples were collected from the mouse at 0, 1, 3 and 7 d (3 mouse at each time point) after anesthesia. The serum ALT and AST levels of mouse increased one day after operation and quickly returned to normal 3 and 7 days after operation, respectively (Fig. 2A, B). The liver weight increased rapidly to 63% on the first day and returned to normal on the 7th day (Fig. 2C). Ki67 showed that the rate of liver regeneration increased rapidly on day 1, then gradually decreased, and basically returned to normal on day 7 (Fig. 2D). Simultaneously, oil red staining of liver showed that numerous lipid droplets accumulated in the hepatocytes of mouse at 1 day after operation, which decreased after 3 days and almost returned to normal after 7 days (Fig. 2E).

### Changes in lipid metabolites after hepatectomy in mouse

**Fig. 3:**
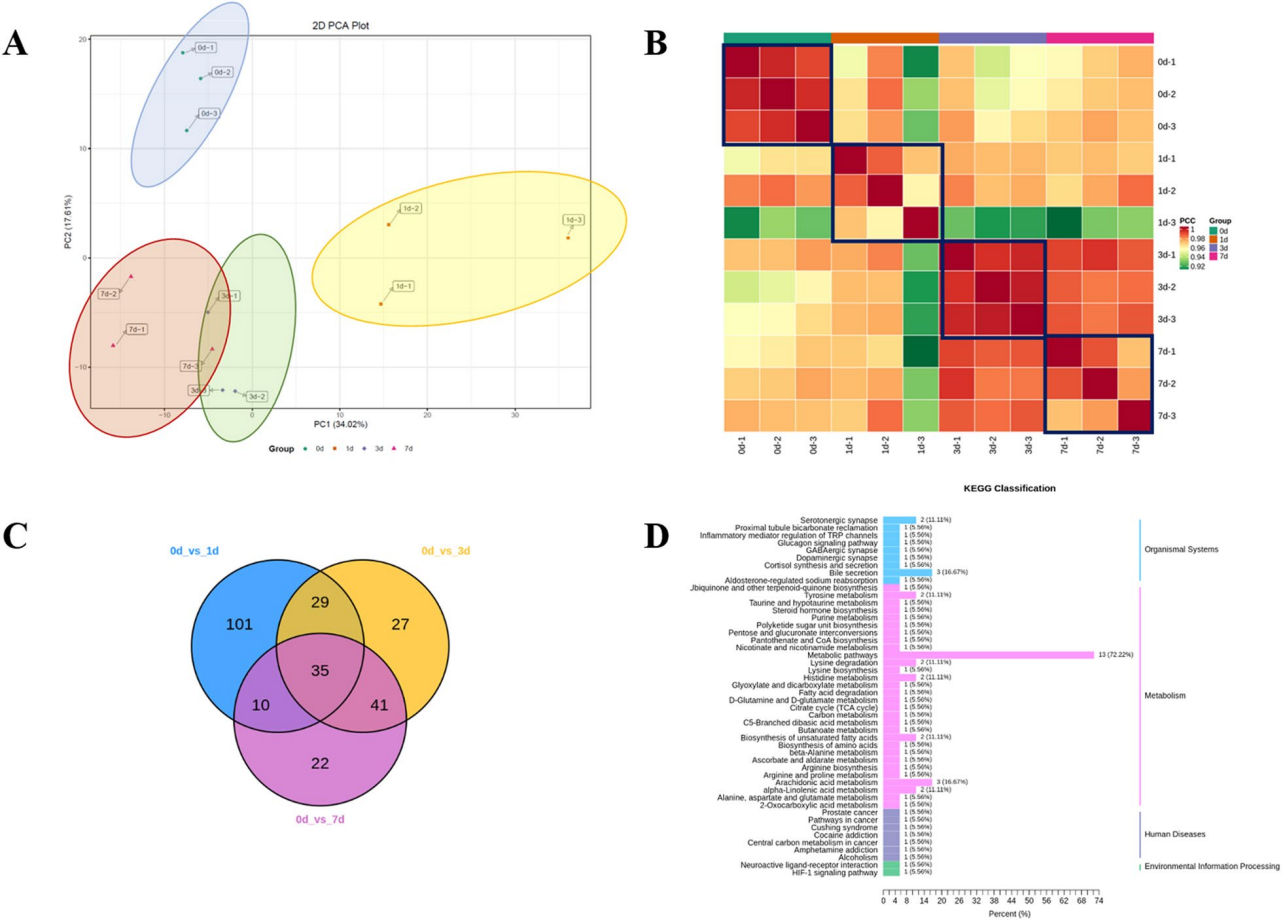
Changes in lipid metabolites after hepatectomy in mouse. **A** We used lipid metabolite sequencing to detect the changes of lipid metabolites at 0, 1, 3 and 7 d after most liver resection in mouse. PCA showed a significant trend of separation among the four groups. **B** Correlation analysis was used to observe within-group repeatability. **C** Pairwise comparison of lipid metabolites at different time points was performed to find the metabolites with differences. In pairwise comparison, VIP >= 1 and absolute log2FC≥1 were used to determine the metabolites that were significantly regulated between groups. **D** KEGG analysis was used to analyze the main pathways of change

We used lipid metabolite sequencing to detect the changes of lipid metabolites at 0, 1, 3 and 7 d (3 mouse at each time point) after most liver resection in mouse. First, the PCA showed a remarkable trend toward intergroup separation among the four groups (Fig. 3A). Second, we performed the correlation analysis to observe the intragroup repeatability, and the results showed high intragroup repeatability (Pearson’s correlation coefficient r^2^ > 0.97) (Fig. 3B). Furthermore, we performed the pairwise comparison analysis to found significantly altered metabolites in liver tissues after hepatectomy. Significantly regulated metabolites between groups were determined by VIP > = 1 and absolute log2FC ≥ 1 in at least two pairwise comparison (Fig. 3C). KEGG analysis results showed that many metabolic pathways were significantly altered (Fig. 3D).

**Fig. 4:**
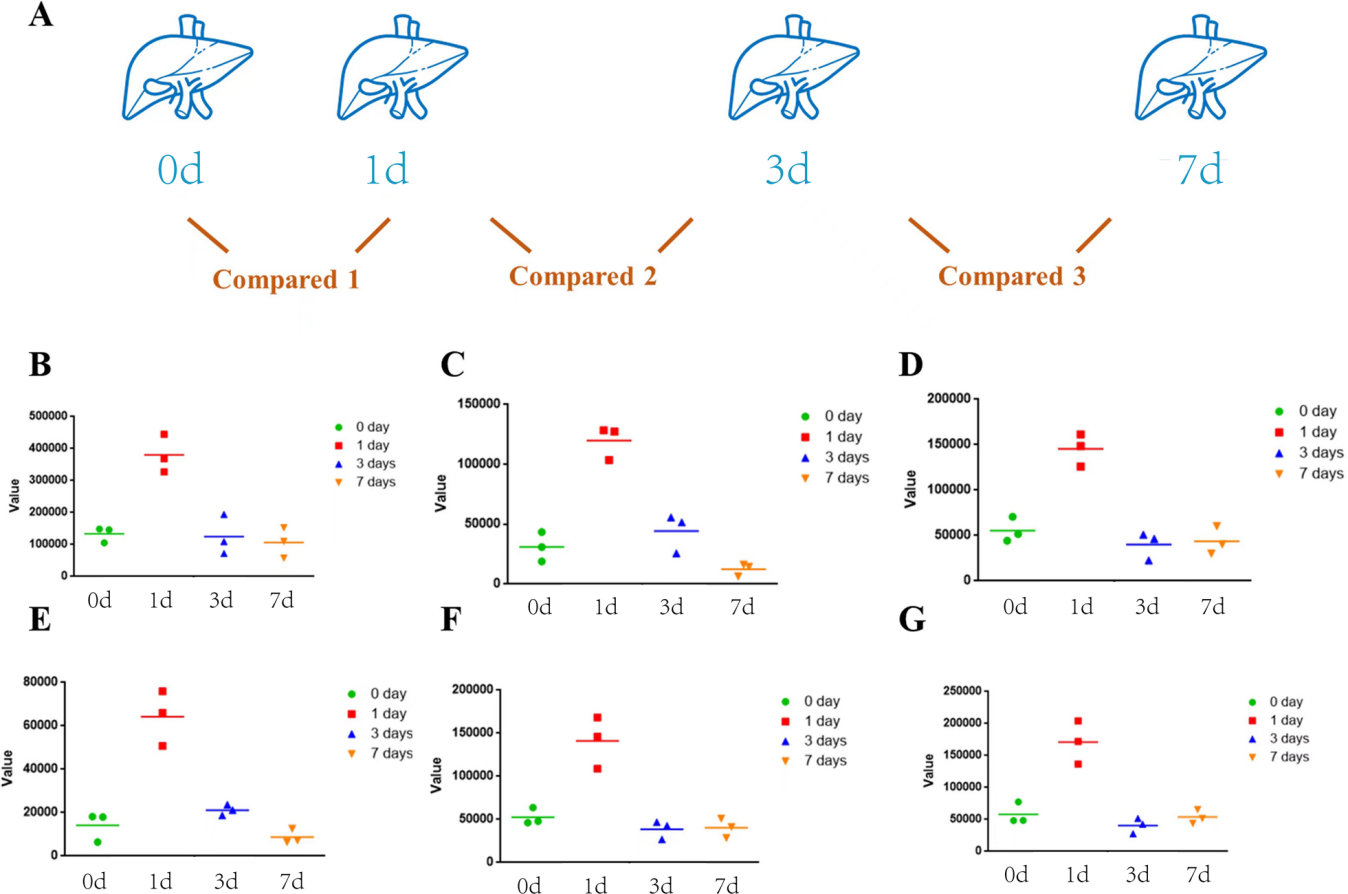
Changes in lipid metabolites following hepatectomy in mouse (pairwise analysis at different time points). **A** Pairwise comparison of lipid metabolite changes. **B–G** According to the same trend of Ki67 in liver regeneration, six lipid metabolites were screened out

To identify metabolites, which were significantly associated with liver regeneration; we further performed the pairwise comparison analysis shown in Fig. 4A. Significantly regulated metabolites between groups were determined by VIP > = 1 and absolute log2FC ≥ 1 in at least two pairwise comparison. Totally 101 altered metabolites were selected and One-way-ANOVA analysis was used to select twelve significantly altered metabolites (*P*_adj_<4.95 × 10^− 4^). After cluster analysis, we finally selected six significantly altered metabolites, which were significantly up-regulated in POD 1 after hepatectomy (Fig. 4B–G), including 2-ethyl-2-hydroxybutyric acid and five types of acylcarnitines (Table 1). Based on these results, we finally selected L-carnitine for further verification.

**Table 1:** Six significantly altered metabolites which were significantly up-expressed in POD 1 after hepatectomy

Index ID	Name of metabolite	Classification
MEDN1098	2-ethyl-2-hydroxybutyric acid	Organic acid and its derivatives
MEDP1084	Dodecylcarnitine	Fatty acyl
MEDP1102	Decanoyl L-Carnitine	Fatty acyl
MEDP1407	Carnitine C14:2	Fatty acyl
MEDP1419	Carnitine C10:0	Fatty acyl
MEDP1518	Carnitine C10:0 Isomer1	Fatty acyl

### L-carnitine can promote liver regeneration after hepatectomy in mouse

**Fig. 5:**
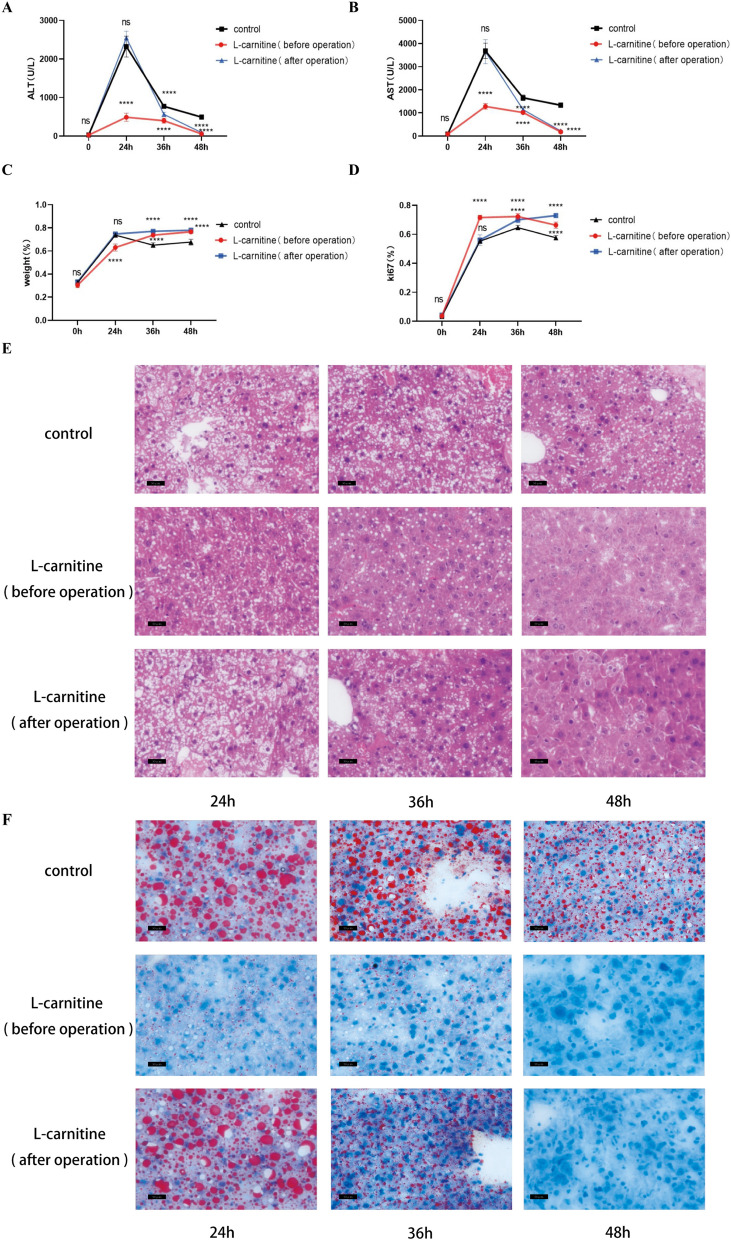
L-carnitine can promote liver regeneration after hepatectomy in mouse. **A**, **B** The liver function indexes (ALT, AST) changed after major partial hepatectomy in mouse in L-carnitine (before operation), L-carnitine (after operation) and control group. **C** The weight changed after major partial hepatectomy in mouse in L-carnitine (before operation), L-carnitine (after operation) and control group. **D** The liver regeneration rate changed after major partial hepatectomy in mouse was measured using Ki67 in L-carnitine (before operation), L-carnitine (after operation) and control group. **E** Oil red staining was used to show the accumulation of lipid droplets in hepatocytes after major partial hepatectomy in mouse in L-carnitine (before operation), L-carnitine (after operation) and control group (400X). **F** HE staining was used to show the basal changes of hepatocytes after major partial hepatectomy in mouse in L-carnitine (before operation), L-carnitine (after operation) and control group (400X). ns p>0.05; ****p<0.0001

We randomly divided the mouse into two groups, one group as the control and the other as the L-carnitine (before operation) group. Serum and liver tissue samples were collected at 24, 36, and 48 h, respectively (There were 3 mouse in each group at each time point). The results showed that the serum ALT and AST of L-carnitine (before operation) group were significantly lower than those of the control group at 3 time points (Fig. 5A, B); in contrast, Ki67 ratio was higher than that of the control group at 3 time points (Fig. 5D). However, the liver weight of L-carnitine (before operation) group was lower than that of the control group at 24 h, and significantly higher than that of the control group at 36 and 48 h (Fig. 5C). HE staining showed that the vacuoles in the L-carnitine (before operation) group were significantly less than those in the control group at 3 time points, and oil red staining also showed that the lipid droplets in the hepatocytes of the L-carnitine (before operation) group were smaller than those in the control group at 3 time points (Fig. 5E, F).

To test whether L-carnitine reduced lipid accumulation in the liver or promoted lipid metabolism in liver regeneration, we administered L-carnitine one day after surgery. Similarly, the serum and liver samples were collected at 24, 36, and 48 h, respectively (There were 3 mouse at each time point). The results showed that serum ALT and AST in L-carnitine (after operation) group were significantly lower than those in the control group at 36 and 48 h after operation (Fig. 5A, B), while Ki67 ratio was higher than that in control group at both time points (Fig. 5D). Similarly, liver weight was significantly higher at 36 and 48 h than that in the control group (Fig. 5C). HE staining showed that the vacuoles of hepatocytes in the L-carnitine (after operation) group were significantly less than those in the control group at 36 and 48 h, and oil red staining showed that the lipid droplets of hepatocytes in the L-carnitine (after operation) group were smaller than those in the control group at both time points (Fig. 5E, F).

### Carnitine acyl transferase I inhibitor inhibited L-carnitine in promoting liver regeneration

**Fig. 6:**
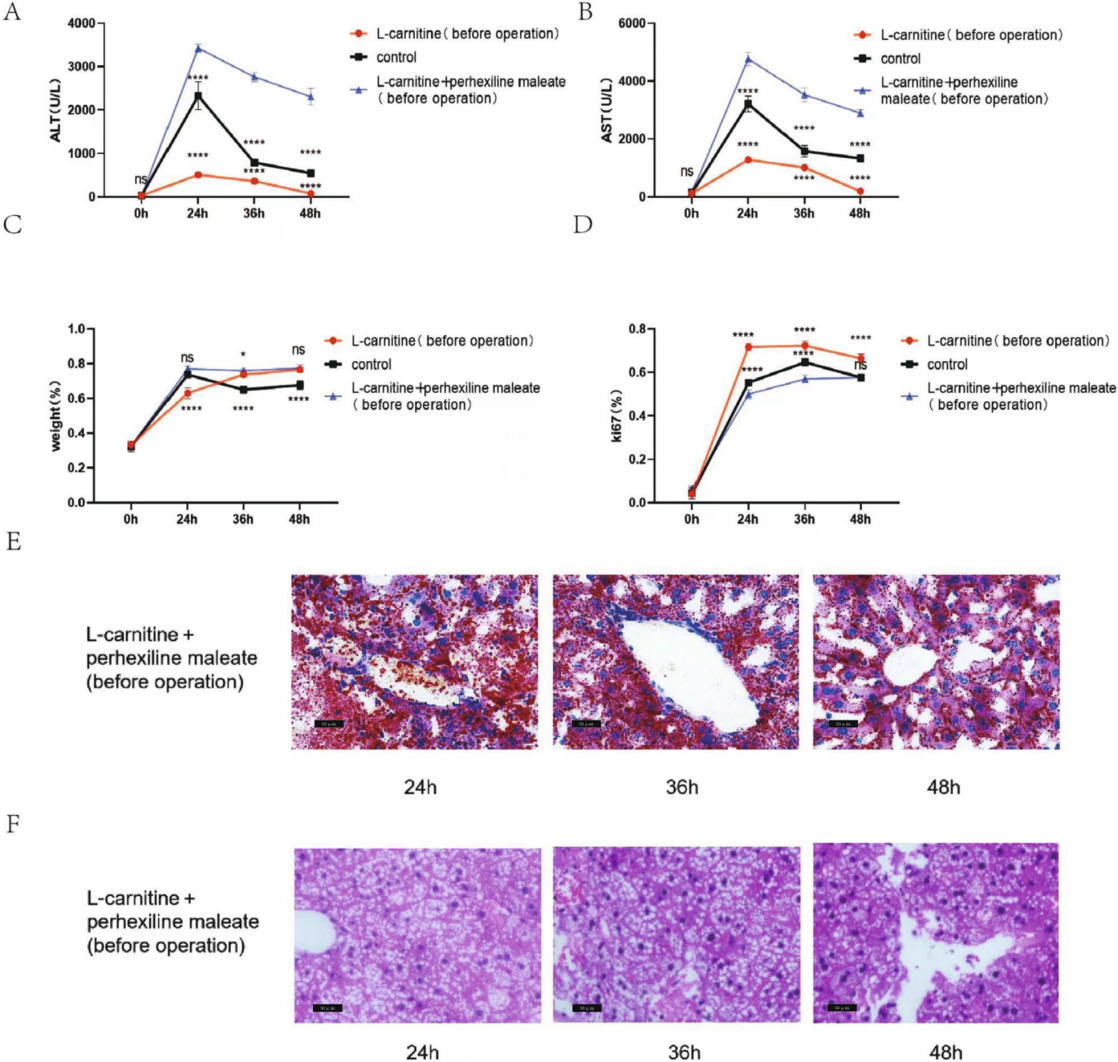
Carnitine acyl transferase I inhibitor inhibited L-carnitine in promoting liver regeneration. **A**, **B** The liver function indexes (ALT, AST) changed after major partial hepatectomy in mouse in L-carnitine (before operation), control and L-carnitine + perhexline maleate group (before operation). **C** The weight changed after major partial hepatectomy in mouse in L-carnitine (before operation), control and L-carnitine + perhexline maleate (before operation) group. **D** The liver regeneration rate changed after major partial hepatectomy in mouse was measured using Ki67 in L-carnitine (before operation), control and L-carnitine + perhexline maleate (before operation) group. **E** Oil red staining was used to indicate accumulation of lipid droplets in hepatocytes after major partial hepatectomy in mouse in L-carnitine + perhexline maleate (before operation) group (400X). **F** HE staining was used to show the basal changes of hepatocytes after major partial hepatectomy in mouse in L-carnitine + perhexline maleate group (before operation) (400X). ns p>0.05; *p<0.05, ***p<0.001, ****p<0.0001

To verify that L-carnitine promotes liver regeneration after hepatectomy in mouse by promoting lipid metabolism, we added carnitine acyl transferase I inhibitor. Serum and liver tissue were collected at 24, 36, and 48 h, respectively (There were 3 mouse at each time point). The results showed that serum ALT and AST in the L-carnitine + perhexiline maleate (before operation) group were significantly higher than those in the L-carnitine (before operation) group and even significantly higher than those in the control group at 3 time points (Fig. 6A, B), while Ki67 ratio was lower than that in the L-carnitine (before operation) group at 3 time points, and also lower than that in the control group at 24 and 36 h (Fig. 6D). The liver weight in the L-carnitine + perhexiline maleate (before operation) group was higher than that in the L-carnitine (before operation) and control groups at 24 h, and it was still higher than that in the control group at 36 and 48 h but slightly lower than that in the L-carnitine (before operation) group (Fig. 6C). However, HE staining showed that the vacuoles of hepatocytes in the L-carnitine + perhexiline maleate (before operation) group were significantly higher in number than those in the L-carnitine (before operation) group and the control group at 3 time points. Oil red staining showed that the lipid droplets of hepatocytes in the L-carnitine + perhexiline maleate (before operation) group were significantly higher than those in the L-carnitine (before operation) group and the control group at 3 time points (Fig. 6E, F).

**Fig. 7:**
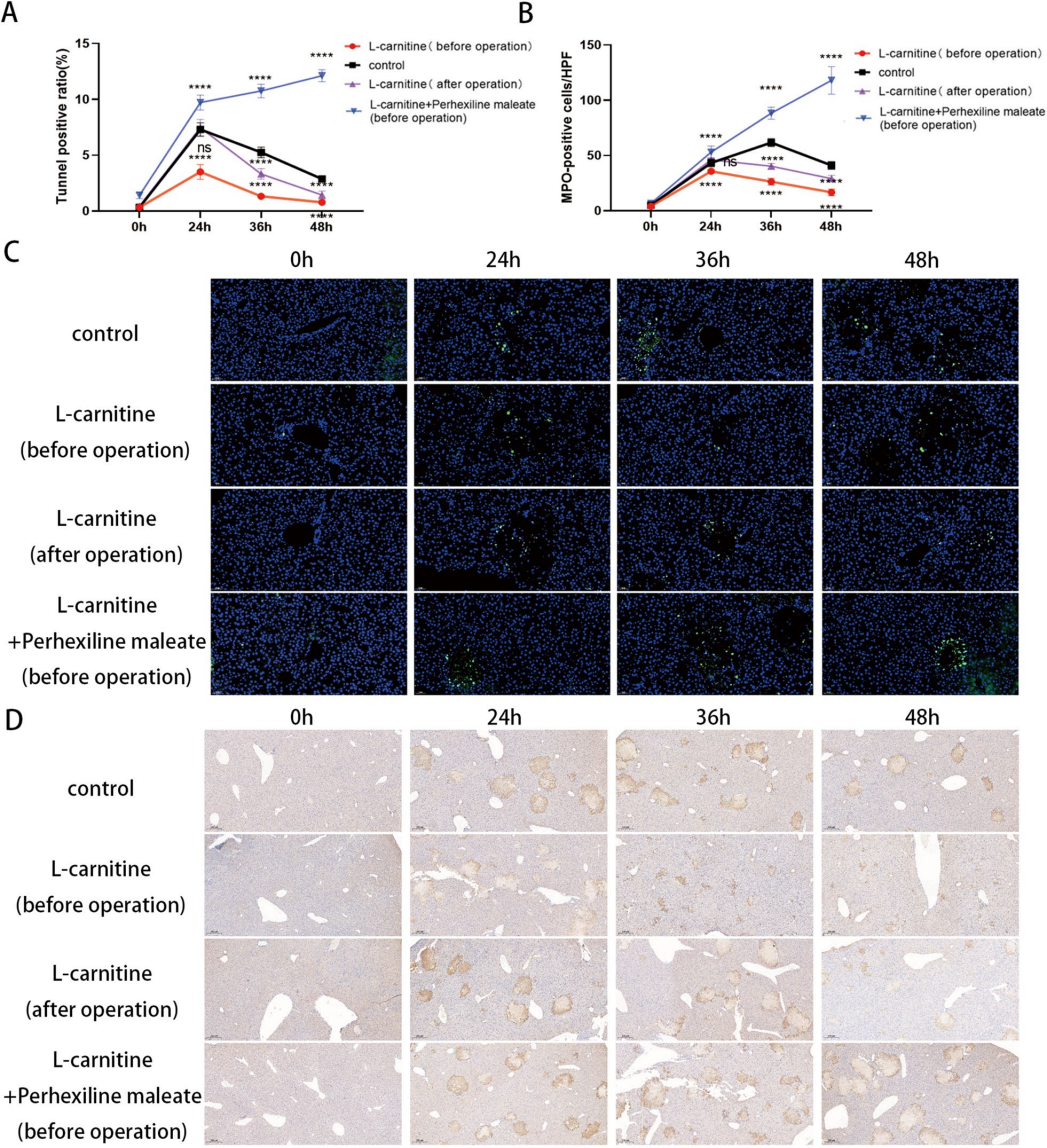
Effects of L-carnitine in liver regeneration on the apoptosis and inflammation. **A **and **C**, Apoptosis of hepatocytes after major partial hepatectomy in mouse in L-carnitine (before operation), L-carnitine (after operation), control and L-carnitine + perhexline maleate (before operation) group was detected by Tunnnel staining (10X). **B** and **D**, Liver inflammation after major partial hepatectomy in mouse in L-carnitine (before operation), L-carnitine (after operation), control and L-carnitine + perhexline maleate (before operation) group was detected by MPO staining (5X). ns p>0.05; ****p<0.0001

Finally, TUNNEL staining and MPO staining were used to detect liver apoptosis and inflammation in the control group, L-carnitine group (before operation), L-carnitine group (after operation) and L-carnitine + perhexiline maleate (before operation) group. The results showed that the liver apoptosis in the L-carnitine + perhexiline maleate (before operation) group was significantly higher than that in the control group at 3 time points, while the L-carnitine (before operation) group was significantly lower than that in the control group. The L-carnitine (after operation) group was significantly lower than the control group at 36 and 48 h (Fig. 7A, C). Similarly, the liver inflammation in the L-carnitine + perhexiline maleate (before operation) group was significantly higher than that in the control group at 3 time points, while the L-carnitine (before operation) group was significantly lower than that in the control group. The L-carnitine (after operation) group was significantly lower than the control group at 36 and 48 h (Fig. 7B, D).

**Fig. 8:**
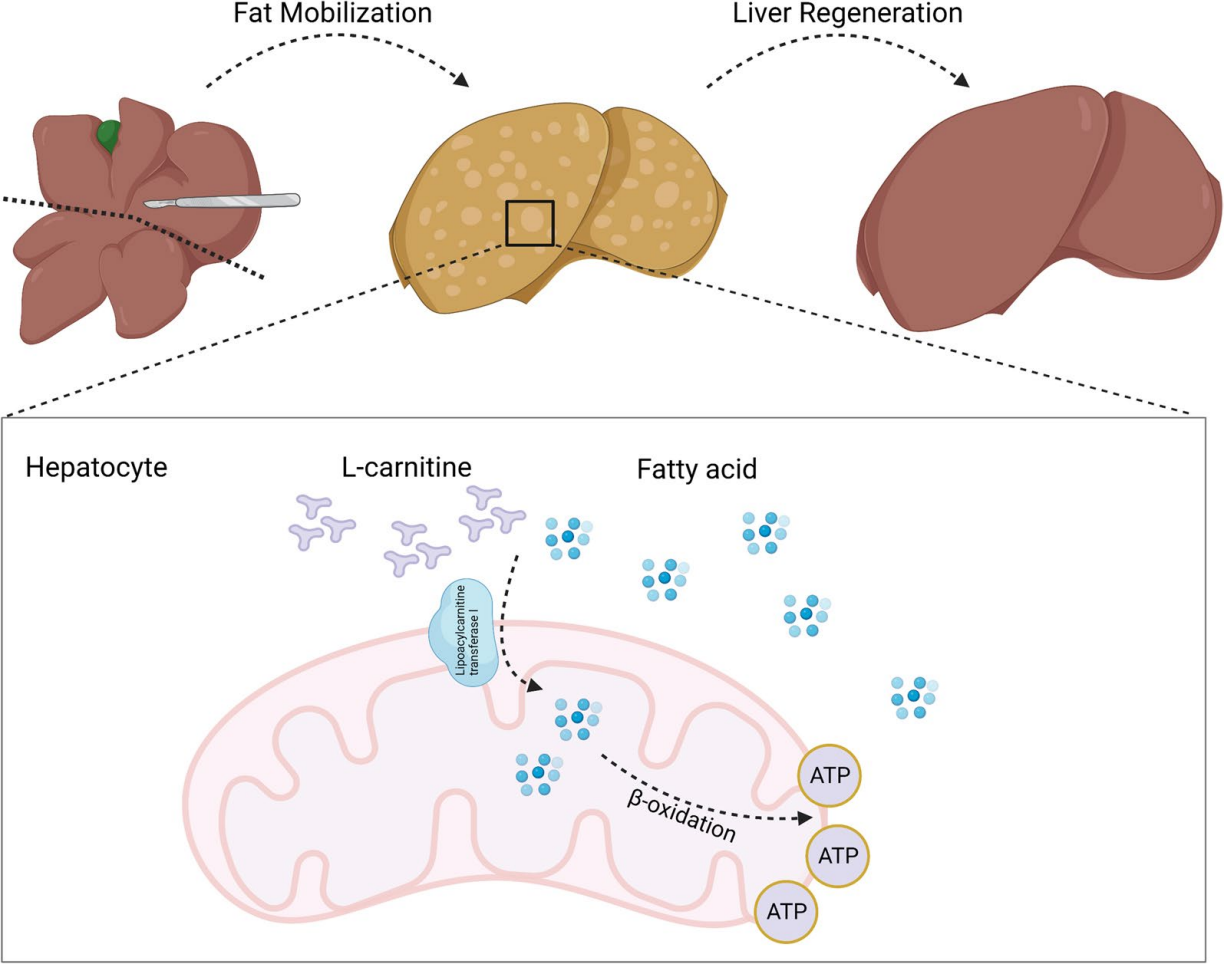
Diagram of L-carnitine's mechanism of promoting liver regeneration in mouse after hepatectomy. In the remaining liver after partial hepatectomy, lipid metabolism is enhanced, in which process, a large number of fatty acids enter the hepatocyte. A higher level of L-carnitine facilitates the transport of fatty acids into mitochondria via carnitine acyltransferase I, where they are oxidized to provide ATP and energy for liver regeneration

Taken together, L-carnitine’s mechanism of promoting liver regeneration in mouse after hepatectomy appears to be through promoting lipid metabolism (Shown in Fig. 8).

## Discussion

As the most critical organ of lipid metabolism, most of the synthesis and utilization of lipid acids occur in the liver [22]. Various liver diseases are also closely associated with lipid metabolism disorders, including nonalcoholic fatty liver disease and hepatic ischaemia/reperfusion injury [23, 24]. Lipid metabolism plays an important role in liver regeneration. Our study showed that after majority (70%) of the mouse liver was removed, the weight of the mouse liver could be restored to normal on the 7th day after resection. Ki67 staining showed that the liver regeneration capacity reached the maximum value at approximately 24 h after the operation and decreased to the normal level after 7 days. Oil red staining showed that the stain first accumulated in hepatocytes after liver resection, reached the peak at 24 h, and then gradually decreased for 7 days and returned to the normal level. We hypothesized that after liver resection, lipids first accumulate in liver cells, and then the liver uses lipid β-oxidation for energy supply. This study also verified that lipid β-oxidation provides energy for liver regeneration after hepatectomy [25]. Therefore, we detected the changes of lipid metabolites during liver regeneration. As expected, a large number of lipid metabolites changed during liver regeneration, and 12 lipid metabolites showed statistically significant changes during liver regeneration. Five of them are lipoacylcarnitine, which is directly proportional to the rate of liver cell proliferation during liver regeneration. Lipoacylcarnitine is an intermediate of lipid β-oxidation and represents the rate of lipid metabolism.

A molecule involved in fatty acid metabolism, L-carnitine is synthesized in the human body using the amino acids L-lysine and L-methionine. L-carnitine is found in many foods, but red meat, such as beef and lamb, is the best choice to add it to your diet. Good sources of carnitine also include fish, poultry, and milk. Through L-carnitine, fatty acid chains are transported to the mitochondrial matrix, where they are broken down and used for energy. Recent studies have begun to elucidate that L-carnitine has beneficial effects in a variety of clinical treatments. L-carnitine and its esters, which can help reduce oxidative stress, have been suggested as treatments for various conditions, such as heart failure, angina, and weight loss [26]. O Parlak et al. found that L-carnitine supplementation enhanced liver regeneration after hepatectomy in rats [27]. Carnitine can promote liver lipid utilization by promoting lipid autophagy [28]. We first treated mouse with L-carnitine before hepatectomy, and found that L-carnitine could significantly reduce the serum ALT and AST contents of mouse, promote the regeneration of hepatocytes, and reduce the lipid deposition of hepatocytes, but the liver weight of L-carnitine group was lower than that of the control group at 24 h. After we think in most of the mouse liver resection, the stress as a result of the surgery, peripheral fat mobilization and serum ALT, AST causes residual liver cells increased burden, gathered a large number of lipid in liver cell formation of lipid droplets, the lipid on the one hand, can give a short period through the beta oxidation liver regeneration process that require large amounts of energy to provide energy, In contrast, excessive accumulation of lipid droplets will cause aseptic inflammation to hepatocytes and damage liver function, which is a double-edged sword. In the control group, we can find that the liver rapidly grows from 30% weight to 60% weight in 24 h. However, this weight does not represent the hepatocyte regeneration, but merely the absorption of a large amount of fat in the liver. This hypothesis can be verified by HE staining and oil red staining. L-carnitine can promote the utilization of lipids, thus providing more energy for liver regeneration. We can observe that the regeneration rate of hepatocytes in L-carnitine group was significantly increased, and liver function was also significantly enhanced. We believe that L-carnitine promotes fat metabolism and leads to the reduction of lipid droplets in hepatocytes.

However, it is unknown whether L-carnitine reduces the accumulation of lipids in the liver or accelerates the metabolism of lipids in the liver. Therefore, we treated mouse with L-carnitine from one day after subtotal hepatectomy, and the results showed that lipid droplets accumulated in numerous hepatocytes at 24 h. HE staining and oil red staining showed that the reduction rate of lipid droplets in the L-carnitine (after operation) group was significantly faster than that in the control group, indicating that L-carnitine indeed accelerated the lipid metabolism in the liver. Simultaneously, postoperative Ki67 staining of L-carnitine (after operation) group showed that compared with the control group, the liver regeneration ability was significantly enhanced, suggesting that L-carnitine promoted liver regeneration by promoting hepatocyte lipid metabolism. The principle of carnitine is to transport long chain fat across the cell membrane into the carrier of mitochondrial oxidation and decomposition to provide energy for the body, to catabolize fat. To confirm the relationship between lipid metabolism and liver regeneration, we further added inhibitors of carnitine acyltransferase I, a key enzyme in lipid metabolism. The results indicating that the perhexiline maleate significantly inhibited the metabolism of lipid in hepatocytes and thus affected liver regeneration. However, the weight of the liver was higher than that of the control group, which was considered due to the accumulation of numerous lipid droplets in the liver after the analysis of HE staining and oil red staining. Although the weight of the liver increased, the hepatocytes did not divide and regenerate and the liver function remained very low. Then we examined inflammation in the liver and found that L-carnitine reduced inflammation in the liver while perhexiline maleate increased inflammation, and we analyzed that the accumulation of lipids in the liver after hepatectomy has a negative effect of damaging hepatocytes along with providing energy. L-carnitine can accelerate the lipid metabolism in hepatocytes and reduce the lipid droplets in hepatocytes, thereby reducing the damage of lipid droplets to hepatocytes. However, our study did not elucidate how lipid droplets affect inflammation and apoptosis during liver regeneration, which will be further investigated in the future.

## Conclusions

In conclusion, our study confirmed the importance of lipid metabolism in liver regeneration after hepatectomy and found that L-carnitine could promote liver regeneration by promoting lipid metabolism after hepatectomy. It provides some theoretical basis and clinical guidance for liver regeneration after hepatectomy, relative liver transplantation and small liver transplantation.

## Data Availability

All data included in this study are available upon request by contact with the corresponding author.

## Abbreviations

ALT: Alanine transaminase
AST: Aspartate transaminase
RPM: Revolutions per minute
H&E: Hematoxylin-eosin
LC-MS/MS: Liquid chromatography coupled to tandem mass spectrometry (LC-MS/MS)
QQQ: Triple quadrupole
QTRAP: A triple quadrupole-linear ion trap mass spectrometer
KEGG: Kyoto encyclopedia of genes and genomes
MSEA: Metabolite sets enrichment analysis
MPO: Myeloperoxidase
PCA: Principal component
HCA: Hierarchical cluster analysis
PCC: Pearson’s correlation coefficients
POD: Postoperative day
